# Development and validation of a deep learning model for detecting signs of tuberculosis on chest radiographs among US-bound immigrants and refugees

**DOI:** 10.1371/journal.pdig.0000612

**Published:** 2024-09-30

**Authors:** Scott H. Lee, Shannon Fox, Raheem Smith, Kimberly A. Skrobarcek, Harold Keyserling, Christina R. Phares, Deborah Lee, Drew L. Posey

**Affiliations:** 1 National Center for Emerging and Zoonotic Infectious Diseases, US Centers for Disease Control and Prevention, Atlanta, Georgia, United States of America; 2 G2S Corporation, San Antonio, Texas, United States of America; Mayo Clinic Arizona, UNITED STATES OF AMERICA

## Abstract

Immigrants and refugees seeking admission to the United States must first undergo an overseas medical exam, overseen by the US Centers for Disease Control and Prevention (CDC), during which all persons ≥15 years old receive a chest x-ray to look for signs of tuberculosis. Although individual screening sites often implement quality control (QC) programs to ensure radiographs are interpreted correctly, the CDC does not currently have a method for conducting similar QC reviews at scale. We obtained digitized chest radiographs collected as part of the overseas immigration medical exam. Using radiographs from applicants 15 years old and older, we trained deep learning models to perform three tasks: identifying abnormal radiographs; identifying abnormal radiographs suggestive of tuberculosis; and identifying the specific findings (e.g., cavities or infiltrates) in abnormal radiographs. We then evaluated the models on both internal and external testing datasets, focusing on two classes of performance metrics: individual-level metrics, like sensitivity and specificity, and sample-level metrics, like accuracy in predicting the prevalence of abnormal radiographs. A total of 152,012 images (one image per applicant; mean applicant age 39 years) were used for model training. On our internal test dataset, our models performed well both in identifying abnormalities suggestive of TB (area under the curve [AUC] of 0.97; 95% confidence interval [CI]: 0.95, 0.98) and in estimating sample-level counts of the same (-2% absolute percentage error; 95% CIC: -8%, 6%). On the external test datasets, our models performed similarly well in identifying both generic abnormalities (AUCs ranging from 0.89 to 0.92) and those suggestive of TB (AUCs from 0.94 to 0.99). This performance was consistent across metrics, including those based on thresholded class predictions, like sensitivity, specificity, and F1 score. Strong performance relative to high-quality radiological reference standards across a variety of datasets suggests our models may make reliable tools for supporting chest radiography QC activities at CDC.

## Author summary

After COVID-19, tuberculosis is the second leading cause of death from infectious disease in the world. The U.S. has relatively low rates of the disease—about 2.5 cases per 100,000 members of the population in 2022—but immigrants, refugees, and other migrants seeking entry into the U.S. often come from areas where the background rates are much higher. To help treat these populations and prevent disease from being imported into the U.S., the Division for Global Migration Health (DGMH) at the Centers for Disease Control and Prevention (CDC) oversees a health screening program for applicants seeking entry. To help detect tuberculosis, most of these applicants receive a chest x-ray, which is then checked for signs of the disease. DGMH receives around half a million of these x-rays each year and conducts ad-hoc quality control assessments to make sure the panel physicians overseas are interpreting the x-rays according to the screening program’s standards. To make these assessments more efficient, we developed a machine learning algorithm that can reliably detect signs of tuberculosis in the x-rays. In testing, the algorithm worked well on a variety of datasets, suggesting it will be a good tool for supporting these important quality control efforts.

## Introduction

Tuberculosis is an infectious disease caused by *Mycobacterium tuberculosis* (MTB) that typically affects the lungs [1]. Those who are infected but do not show symptoms have latent tuberculosis infection (LTBI) and may never develop tuberculosis disease. LTBI is not infectious but still needs to be treated to prevent the progression into tuberculosis disease. Tuberculosis disease causes coughing, chest pain, fatigue, weight loss, fever, and many other symptoms, and is contagious [2]. It is the 13^th^ leading cause of death in the world, and the second leading infectious killer after COVID-19 [1]. In the United States, tuberculosis rates have been declining, and the tuberculosis incidence rate for 2021 was 2.4 cases per 100,000 persons, with the majority of reported cases occurring among non-US–born persons (71.4%). Non-US born persons had an incidence rate 15.8 times higher (12.5 cases per 100,000) when compared to US-born persons (0.8 cases per 100,000) [3].

Every year, approximately 550,000 immigrants and refugees apply to enter the United States. The Division of Global Migration Health (DGMH) within the Centers for Disease Control and Prevention (CDC) has regulatory responsibility to oversee the medical examinations of these applicants. The examinations are conducted overseas in accordance with CDC DGMH’s Technical Instructions for panel physicians. All panel physicians are licensed local medical doctors on an agreement with the US Department of State to perform these examinations, and many are affiliated with the International Organization for Migration (IOM), an intergovernmental agency under the United Nations system that supports migrants. IOM works closely with US Department of State and CDC to ensure the healthy migration of US-bound immigrants and refugees.

DGMH’s Technical Instructions for tuberculosis seek to prevent disease importation by detecting and treating infectious tuberculosis before arrival, and to reduce tuberculosis-related morbidity and mortality in these populations. Requirements include a medical history and physical examination. All applicants 15 years and older receive chest x-rays, and anyone with a chest x-ray suggestive of tuberculosis, signs or symptoms suggestive of tuberculosis, or known HIV, then has three sputum specimens collected for smears and cultures [4–5]. In September 2018, DGMH began receiving digital copies of chest x-ray images from panel sites. This was due to the rollout of the eMedical system, an electronic health processing system that collects data from the required overseas immigrant examinations. In 2018 alone, 124,551 images for 521,270 applicants were collected, raising the possibility of using machine learning methods to complement DGMH’s already effective oversight for the radiologic components of tuberculosis screening for US-bound immigrants and refugees [6].

### Brief overview of AI for Chest radiography

Artificial intelligence (AI), especially as enabled by deep learning algorithms, has been widely studied for applications in medical imaging. Examples include diabetic retinopathy [7], cardiovascular risk prediction [8], cancer histopathology [9–11], and imaging for musculoskeletal [12–13], cardiac [14], and pulmonary [15] conditions. Models are typically designed for diagnostic tasks, like segmenting anatomical structures or indicating the presence of disease, but they have also been designed for prognostic tasks, like predicting survival time for patients from histopathology whole-slide images [16].

In chest imaging, applications have generally focused on identifying abnormalities associated with specific diseases, like pneumonia [17–18], COVID-19 [19], lung cancer [20–21], and tuberculosis [15,17,22]^.^ Recent work [23–24] has broadened the scope to include abnormalities in general. Studies focusing on tuberculosis have ranged from the narrow evaluation of specific models (typically commercial) on relatively small test sets [25–26] to the development of original algorithms from custom largescale training sets [23,27–28]. The references standards for these studies are often mixed, comprising radiological findings, clinical diagnoses, microbiological testing, and nucleic acid amplification testing (NAAT).

Of special note, when laboratory tests are used as reference standards, model performance tends to drop relative to performance against a radiological standard; however, a small number of models have met the World Health Organization’s (WHO) Target Product Profile (TPP) for tuberculosis triage tests at 90% sensitivity and at least 70% specificity [26,29] relative to NAAT or culture, even when testing does not rely on initial radiographic interpretation to identify images with abnormalities (see e.g. Qin et al. [26] and Khan et al. [30], where all study participants received both a chest x-ray and either a GeneXpert MTB/RIF test or a sputum culture upon enrollment).

### Project goal

The primary use-cases of models in the literature have mostly been clinical decision support and workflow improvement, with special emphasis on individual-level classification performance (often as measured by AUC), interpretability, and usability. With respect to TB, emphasis has also been placed on the potential benefit for models to bolster TB screening and diagnosis in low-resource settings, e.g., by rank-ordering radiographs in batches by their probability of disease to guide manual review. For this project, we evaluated our models’ ability to meet these goals, and we also sought to evaluate their performance in estimating sample-level prevalence, i.e., in predicting the number of abnormal x-rays in a given batch. These measures mirror two important operational use-cases of the model in the overseas screening program: supporting panel physicians in providing high-quality initial reads during the exams (an unplanned but potentially impactful application), and enabling DGMH to conduct quality control (QC) with the radiographs once they have been collected (the primary focus of our current project).

To achieve our goals, we trained and validated models for performing three tasks: classifying images as abnormal (Task 1), classifying images as abnormal and suggestive of tuberculosis (Task 2), and identifying the specific abnormalities in the images (Task 3) (we use the same numbering scheme to identify the corresponding models). To meet the two use-cases above, we tested our models on a variety of data sets, both internal and external, and we measured their performance using two operating points, one chosen to optimize individual-level classification performance, and one chosen to optimize accuracy in predicting prevalence. Although we did not formally test abnormality localization methods, e.g., via object detection models, we implemented a number of common saliency methods for visualizing suspected abnormalities on the input images to improve model interpretability and pilot interactive methods for manual review.

## Methods

### Internal dataset curation and description

For our internal datasets (hereafter HaMLET, from our project title, Harnessing Machine Learning to Eliminate Tuberculosis), we obtained an initial convenience sample of 327,650 digitized radiographs from four sources: eMedical, the US Department of State’s immigrant health data system, a web-based application for recording and transmitting immigrant medical cases between the Panel Physicians, US Department of State, and the CDC [31]; the Migrant Management Operational System Application (MiMOSA), the International Organization for Migration’s (IOM) refugee health data system; IOM’s Global Teleradiology and Quality Control Centre (GTQCC); and a small number of individual US immigrant panel sites that screen a relatively high number of applicants with tuberculosis each year (site names are provided in the Acknowledgments). Importantly, all these sites have experienced radiologists, and most conduct either double or triple readings on all chest x-ray images as a measure of quality control. Regardless of source, all radiographs were stored as Digital Imaging and Communications in Medicine (DICOM) files, and all radiographic findings were extracted directly from the structured entries in the DS-3030 Tuberculosis Worksheet [32] instead of from free-text radiology reports by way of natural language processing (NLP).

**Fig 1:**
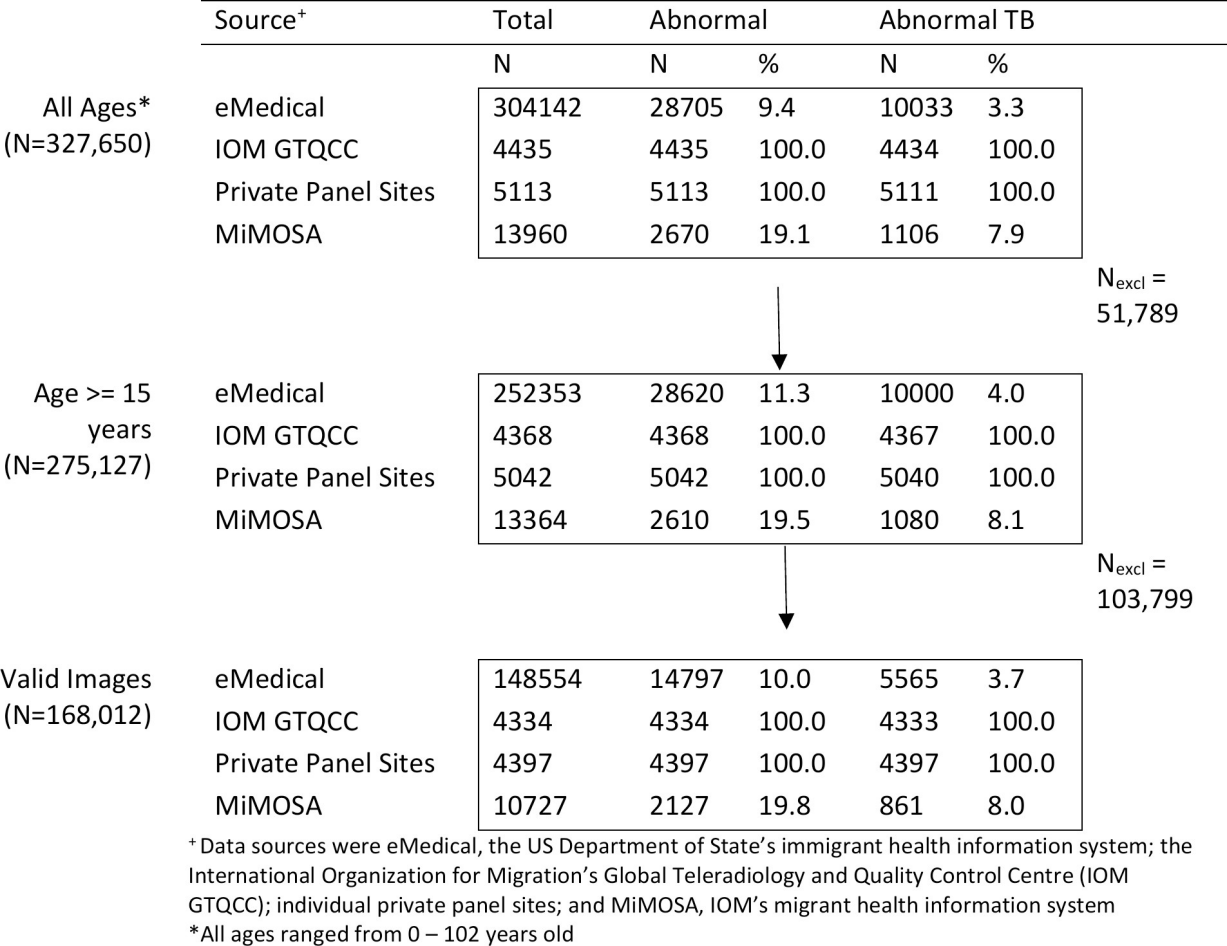
Flow diagram detailing the number of radiographs by data source and abnormality status from before exclusion for age (All Ages), after exclusion for age under 15 years, and after exclusion for whether the DICOM pixel arrays were Python-readable (Valid Images). Radiographs were collected between the years of 2011 and 2021 and constitute a convenience sample of the total applicant population for that time period. The number of images excluded at each step is shown in the last column as N_excl_.

The set assembled for this project was taken from screenings conducted during a ten-year period from October 2011 to October 2021 and not exclusively from the digitized radiographs routinely received by DGMH since 2018 (S1 Table shows the distribution of exams by region and year). The digitized radiographs began in 2018 due to the eMedical rollout, but we also received screenings directly from private immigrant panel sites and from IOM that predated the eMedical rollout. We excluded radiographs from applicants less than 15 years of age (n = 52,523), as well as those stored in DICOM files whose pixel arrays were missing, corrupt, or otherwise unreadable by the software we used for extraction (n = 107,115) (Fig 1 shows a flow diagram providing a detailed numerical accounting of these two exclusion steps). The remaining 168,012 radiographs constituted our final dataset, which we split into training, validation, and testing portions following the procedure described below.

### Radiologist annotations

Chest radiograph abnormalities for the immigration exam fall into one of two groups: those suggestive of tuberculosis and those not. Abnormalities suggestive of tuberculosis include: infiltrates or consolidations; reticular markings suggestive of fibrosis; cavitary lesions; nodules or mass with poorly defined margins; pleural effusions; hilar or mediastinal adenopathy; miliary findings; discrete linear opacities; discrete nodule(s) without calcification; volume loss or retraction; and irregular thick pleural reaction. Abnormalities not suggestive of tuberculosis include cardiac, musculoskeletal, or other abnormalities; smooth pleural thickening; diaphragmatic tenting; calcified pulmonary nodules; and calcified lymph nodes.

Most abnormal images in our internal validation and test sets were suggestive of tuberculosis. Although we did benchmark our generic model against two open datasets with a wider range of abnormalities (described below), we focused primarily on the tuberculosis classification task for our analysis. Importantly, however, because only a small number of the abnormal images (1,551) were from applicants with active tuberculosis at the time of screening—the vast majority were from applicants who had previously been screened, diagnosed with tuberculosis, and received treatment, or whose tuberculosis sputum testing results were negative—we chose not to benchmark our models against a microbiological or bacteriologic reference standard, focusing instead on a purely radiological reference standard.

### External test sets

To supplement our internal testing data, we benchmarked our binary models 1 and 2 on four external datasets: ChestX-ray8 [33]; the Montgomery County, USA (MCU) and the Shenzhen, China (SHN) tuberculosis datasets [34]; and VinDr-CXR [35]. VinDr-CXR was the largest with 2,971 images in total, 161 of which were suggestive of tuberculosis; the others ranged in size from 138 images (MCU) to 810 images (ChestX-ray8). For all datasets, we use the testing splits specified in their original publications, and the original labels, with the exception of ChestX-ray8, for which we use the refined test labels provided by Google [23,27].

MCU, SHN, and VindDr-CXR had labels indicating the suggested presence of tuberculosis (reference standards varied by dataset and included radiographic, clinical, and laboratory evidence of disease), but only ChestX-ray8 and VinDr-CXR also had labels indicating a variety of other abnormalities. Because of this imperfect overlap between our classification tasks and the labels in the datasets, VinDr-CXR is the only dataset on which we test both binary models (1 and 2); for the other three, we test only Model 1 (ChestX-ray8) or Model 2 (MCU and SHN).

### Dataset splitting

For our internal data, we began with 168,012 images in total, which we then randomly split into training (152,012; 15% abnormal), validation (8,000; 50% abnormal), and testing (8,000; 50% abnormal) sets, following a sample size calculation we used to determine the number of images we would need to achieve a 5% margin of error in estimating sensitivity (technical details on the procedure are provided in the S1 Text). Training images were single-read images randomly drawn from all sites. Testing and validation images for Task 2 had either been double-read as part of the IOM Teleradiology QA/QC program or single-read at a handful of panel sites in areas with high TB burden.

For ChestX-ray8, we reserved an additional 8,000 images from the original training data to serve as validation data for Task 1 (in our internal validation dataset, abnormalities not suggestive of tuberculosis were underrepresented, as the abnormal images were almost always abnormal and suggestive of tuberculosis).

### Operating point selection

When validation data was available, we used it to select two operating points for thresholding the models’ predictions on the corresponding test sets: one that maximized Youden’s J index (all tasks), and one that minimized the relative error in predicted prevalence (Tasks 2 and 3 only). We named these two operating points the “J” and “count” operating points, respectively. Because the proportion of abnormal images in our internal test set was different than the corresponding proportion in the training set, the latter being generally representative of the screening program’s data distribution over a multiyear period of time, the count-based operating points were selected using a reweighting scheme that minimized error in predicting the proportion from the training set using the model’s performance characteristics (sensitivity and specificity) on the validation set; this procedure is described in full in S1 Text. Finally, when validation data was not available, as was the case for all external datasets except for ChestX-ray8, we selected a single operating point that maximized Youden’s J index on the test sets. We provide all operating points in S2 Table.

### Image preprocessing

After discarding DICOM files with corrupt pixel arrays, we extracted the pixel arrays and saved them as 1024x1024-pixel PNG files. We then used optical character recognition (OCR) software to identify images with evidence of burned-in patient metadata and removed them from the dataset. We describe both of these procedures more fully in S1 Text.

### Model architecture and training procedures

To improve the model’s ability to generalize to unseen data, we used a custom image augmentation layer as the input layer, randomly perturbing brightness, contrast, saturation, and other characteristics to the radiographs during training; value ranges for these perturbations were taken from Majkowska et al. 2020 and remained fixed during training [27]. For the feature extractor, we used EfficientNetV2M [36], which was pretrained on ImageNet [37]. The final layers in our model were a dropout layer (probability = 0.5, held fixed) and then a dense layer with a sigmoid activation and binary cross-entropy loss.

We trained all models in minibatches of 12 images (4 per GPU) with the Adam [38] optimizer and a fixed learning rate of 1e-4. For all tasks, we allowed training to continue until AUC began to decrease on the validation data at which point we saved the model weights and proceeded to testing.

### Performance metrics and statistical inference

We calculated common classification performance metrics for all models and test sets, including AUC, sensitivity, specificity, and F1. For tuberculosis-specific datasets, we also calculated specificity at 90% sensitivity and sensitivity at 70% specificity, in line with the WHO’s TPP for tuberculosis triage tests for use in community settings. For the HaMLET test set, we calculated the model’s relative error in predicting prevalence (i.e., the true number of abnormal-TB images), mirroring our primary operational use-case for the model as a tool for internal QC activities.

For all metrics, we calculated bias-corrected and accelerated (BCA) bootstrap confidence intervals [39], down-sampling abnormal images in the bootstrap replicates so the percentage of abnormal images in each was equal to the percentage in the training data (target percentage for each task provided in Table 1; see S1 Text for more details). We did not adjust the intervals for multiplicity.

Finally, we note here that model performance may depend not only on the reference standard used for classification (i.e., radiography vs. bacteriologic or molecular testing), but also on the prevalence and severity of abnormalities present in the population in which it is intended to be deployed. Applicants who attend immigration-related health screenings tend to be healthier than, say, patients in inpatient settings or those whose present with severe clinical symptoms of TB, and so we expect certain of these metrics to be lower in our study than they would be in a study of patients with more severe disease.

**Table 1:** Distributions of age and sex for the applicants in our training, validation, and testing datasets, along with the geographic distribution of their corresponding health screening exam sites.

		All		Training		Validation		Testing	
		N	%	N	%	N	%	N	%
	Total	168,012	--	152,012	90	8000	5	8000	5
Age Group	15–24	37837	23	34797	23	1541	19	1499	19
	25–34	40715	24	37549	25	1567	20	1599	20
	35–44	28614	17	26035	17	1268	16	1311	16
	45–54	25581	15	23032	15	1302	16	1247	16
	55–64	19949	12	17581	12	1159	14	1209	15
	> = 65	12029	7	9973	7	1050	13	1006	13
	N/A	3286	2	3044	2	113	1	129	2
Sex	F	92851	55	86364	57	3261	41	3226	40
	M	70826	42	65301	43	2742	34	2783	35
	N/A	4334	3	346	0	1997	25	1991	25
Data Source^+^	IOM GTQCC	4334	3	346	0	1997	25	1991	25
	MiMOSA	10727	6	10727	7	0	0	0	0
	Private Panel Sites	4397	3	385	0	2003	25	2009	25
	eMedical	148553	88	140553	92	4000	50	4000	50
Exam Region*	Africa	29753	18	28854	19	430	5	469	6
	Americas	43296	26	33707	22	4803	60	4786	60
	Asia	77755	46	72722	48	2538	32	2495	31
	Europe	15369	9	14895	10	228	3	246	3
	Oceania	1453	1	1453	1	0	0	0	0
	N/A	385	0	380	0	1	0	4	0
Exam Sub-Region	Australia and New Zealand	1276	1	1276	1	0	0	0	0
	Central Asia	996	1	996	1	0	0	0	0
	Eastern Asia	11634	7	11634	8	0	0	0	0
	Eastern Europe	7884	5	7410	5	228	3	246	3
	Latin America and the Caribbean	38336	23	28747	19	4803	60	4786	60
	Melanesia	172	0	172	0	0	0	0	0
	Northern Africa	4883	3	4882	3	0	0	1	0
	Northern America	4960	3	4960	3	0	0	0	0
	Northern Europe	1204	1	1204	1	0	0	0	0
	Polynesia	5	0	5	0	0	0	0	0
	South-eastern Asia	34368	20	30552	20	1912	24	1904	24
	Southern Asia	21561	13	20452	13	564	7	545	7
	Southern Europe	5020	3	5020	3	0	0	0	0
	Sub-Saharan Africa	24870	15	23972	16	430	5	468	6
	Western Asia	9196	5	9088	6	62	1	46	1
	Western Europe	1261	1	1261	1	0	0	0	0
	N/A	385	0	380	0	1	0	4	0

+IOM GlobalGlobal Teleradiology and Quality Control Center (GTQCC) and the private panel sites all have their own internal quality control processes.

*Exam Region:

**Africa:** Algeria, Angola, Benin, Burkina Faso, Burundi, Cameroon, Cape Verde, Chad, Democratic Republic of the Congo, Republic of the Congo, Djibouti, Egypt, Gambia, Ghana, Guinea, Ivory Coast, Kenya, Liberia, Malawi, Morocco, Mozambique, Namibia, Niger, Nigeria, Rwanda, Senegal, South Africa, Sudan, Tanzania, Togo, Tunisia, Uganda, Zambia, and Zimbabwe

**Americas:** Argentina, Barbados, Belize, Bolivia, Brazil, Canada, Chile, Colombia, Costa Rica, Dominican Republic, Ecuador, Guatemala, Guyana, Haiti, Honduras, Jamaica, Mexico, Nicaragua, Panama, Paraguay, Peru, Trinidad and Tobago, and Uruguay

**Asia:** Afghanistan, Azerbaijan, Bahrain, Bangladesh, Cambodia, China, Hong Kong, India, Indonesia, Iraq, Israel, Japan, Jordan, Kazakhstan, Republic of Korea, Kuwait, Kyrgyzstan, Laos, Lebanon, Malaysia, Mongolia, Myanmar, Nepal, Oman, Pakistan, Philippines, Saudi Arabia, Singapore, Sri Lanka, Taiwan, Tajikistan, Thailand, Turkey, Turkmenistan, United Arab Emirates, Uzbekistan, Vietnam, and West Bank

**Europe:** Albania, Austria, Belarus, Belgium, Bosnia and Hercegovina, Bulgaria, Croatia, Cyprus, Denmark, Estonia, Finland, France, Georgia, Germany, Greece, Hungary, Italy, Kosovo, Latvia, Lithuania, Malta, Moldova, Netherlands, North Macedonia, Norway, Poland, Portugal, Romania, Russia, Serbia, Slovak Republic, Slovenia, Spain, Sweden, Switzerland, Ukraine, and United Kingdom

**Oceania:** Australia, Fiji, New Zealand, Papua New Guinea, and Tonga

### Abnormality localization

We used two saliency methods, Grad-CAM [40] and XRAI [41], to generate abnormality heatmaps for the images. We examined a small selection of the heatmaps for true-positive and false-positive images (abnormal and normal images, respectively, with high model-based probabilities of abnormality) to explore their use as approximate abnormality localization methods. Because we did not have ground-truth bounding box annotation for the images, this step was primarily exploratory.

### Software and Hardware

Our code is publicly available at https://github.com/cdcai/hamlet.git. Complete information on the software and hardware used is available in S1 Text.

### Ethical considerations

This project was proposed, reviewed, and approved in accordance with CDC institutional review policies and procedures. Because it received a non-research determination, review by an institutional review board was not required. Neither trained model weights nor raw images will be made publicly available to protect applicant privacy. Requests for data may be made through CDC’s Migration Health Information Nexus at mhinx@cdc.gov.

## Results

### Demographic characteristics of our study sample

Table 1 shows the demographic characteristics of the applicants in our study sample, and where their screening exams were conducted. Overall, 55% of the applicants were women, and most (64%) were between the ages of 15 and 44. Applicants 65 years and older were the rarest, constituting 7% of both the overall and training data, followed by applicants between 55 and 64 (12%) and those between 45 and 54 (15%). In our validation and test sets, applicants in the youngest age group (15 to 24) were underrepresented (19% in each), while those in the oldest age group (65 and over) were overrepresented (13% in each), relative to the age distribution in both the overall sample and the training data.

Geographically, most of the exams were conducted in Asia (46%), with Southeastern Asia (20%), Southern Asia (13%), and Eastern Asia (7%) being the three primary contributors to the region. By sub-region, most exams were conducted in Latin America and the Caribbean (23%), which was the main contributor to exam volume in the Americas region (26% of exams overall). By contrast, Oceania (1%) and Europe (9%) had the smallest representation by volume. In our validation and test datasets, these percentages changed substantially, with Latin America and the Caribbean contributing 60% of the images to both, and Southeastern and Southern Asia contributing 31%, with the remaining 9% comprising images from Africa (6%) and Europe (3%).

By data source, the majority of our images (88%) came from eMedical, the US Department of State’s immigrant health data system. Of the remaining images, 6% came from MiMOSA, IOM’s health data system, 3% from the IOM Teleradiology QC program, and 3% from our partner panel sites. Although the training dataset skewed heavily toward images from eMedical (92%), the validation and test datasets were evenly split between eMedical (50%) and the IOM Teleradiology QC program (25%) and our partner panels (25%). As mentioned above, the latter two primarily contributed images that were abnormal and suggestive of TB, and so we used eMedical as the source for normal images, noting that these latter images were also drawn from screenings performed by our partner panel sites and not from the system at random.

### Distribution of abnormalities

Table 2 shows the distribution of general and specific findings across our internal training, validation, and test sets. In the training data, 12% of the images were abnormal and 5% were abnormal and suggestive of tuberculosis with 0.1% of images in the latter category from applicants who were either smear- or culture-positive for tuberculosis disease at the time of screening. In the validation and test sets, these percentages changed to 50%, 50%, and 9% respectively, both because we up-sampled abnormal images to increase precision in estimating sensitivity, and because we had requested additional images from smear- or culture-positive applicants from the IOM Teleradiology QC program and our partner panel sites to use for testing.

By specific finding, the most common abnormalities were the discrete linear opacity (2.4% in training; 20% in validation; 20% in testing) and the infiltrate or consolidation (1.4%; 30%; 30%). In the training data, the rarest abnormalities were miliary findings (<1%), cavitary lesions (0.1%), pleural effusions (0.1%), and hilar/mediastinal adenopathy (0.1%), all of which remained rare in the validation and testing data, despite the up-sampling of abnormal images.

**Table 2:** Counts and proportions of radiographic findings suggestive of tuberculosis (TB) in our internal training, validation, and testing datasets.

		All		Training		Validation		Testing	
		N	%	N	%	N	%	N	%
Overall Classification	Abnormal	25655	15.3	17655	11.6	4000	50	4000	50
	Abnormal (Suggestive of TB)	15156	9	7157	4.7	3999	50	4000	50
Specific Findings	Infiltrate or Consolidation	6970	4.1	2187	1.4	2397	30	2386	29.8
	Reticular Findings	2215	1.3	895	0.6	663	8.3	657	8.2
	Cavitary Lesion	455	0.3	130	0.1	164	2.1	161	2
	Nodule or Mass with Poorly Defined Margins	1297	0.8	521	0.3	400	5	376	4.7
	Pleural Effusion	557	0.3	183	0.1	194	2.4	180	2.2
	Hilar/mediastinal Adenopathy	269	0.2	107	0.1	84	1	78	1
	Miliary Findings	20	0	10	0	8	0.1	2	0
	Discrete Linear Opacity	6718	4	3628	2.4	1528	19.1	1562	19.5
	Discrete Nodule(s) Without Calcification	1763	1	819	0.5	473	5.9	471	5.9
	Volume Loss or Retraction	1346	0.8	558	0.4	398	5	390	4.9
	Irregular Thick Pleural Reaction	1317	0.8	513	0.3	401	5	403	5
	Other	891	0.5	406	0.3	236	2.9	249	3.1
TB Status*	SM/CX+ at Prior Exam	9546	5.7	6973	4.6	1287	16.1	1286	16.1
	SM/CX+ at Current Exam	1551	0.9	150	0.1	689	8.6	712	8.9

*Positive TB Status (+) is defined as having positive sputum smear (SM) and/or culture (CX) results at either the latest recorded exam in the screening process (“Current”) or at one of the previous exams in the same (“Prior”).

### Binary classification performance

Table 3 shows the performance metrics for our models on the two binary classification tasks. For Task 2, AUCs were consistently high, ranging from 0.99 (95% CI 0.97, 1.0) on MCU to 0.94 (0.93, 0.95) on VinDr-CXR. Specificity at a sensitivity of 0.90 was similarly high, ranging from 0.83 (0.78, 0.87) on VinDr-CXR to 0.98 (0.89, 1.0) on MCU, although for reasons we provide in the discussion, we do not suggest whether any of these would meet the WHO’s TPP for tuberculosis triage tests. With the count-based operating point, the model also fared well in predicting the number of abnormal images suggestive of tuberculosis in our internal test set, achieving a relative error of only -2% (-8%, 6%).

Performance was similar, although slightly lower, on Task 1. On ChestX-ray8, the model achieved an AUC of 0.92 (0.90, 0.93) and an optimal sensitivity and specificity of 0.82 (0.78, 0.84) and 0.86 (0.81, 0.89); on VinDr-CXR, these numbers were 0.89 (0.88, 0.90), 0.89, (0.87, 0.90), and 0.73 (0.72, 75), respectively. Because we did not have internal testing data for this task, we did not test this model with the count-based operating point.

**Table 3:** Classification results for our abnormal-TB and abnormal models on our internal dataset and on four external datasets.

	Dataset*	Sensitivity	Specificity	PPV	NPV	J	F1	AUC	Spec@90	Sens@70	Rel. Prev. Diff.
Abn. TB	HaMLET	0.89 (0.86, 0.92)	0.93 (0.93, 0.94)	0.36 (0.32, 0.39)	1.0 (0.99, 1.0)	0.82 (0.79, 0.86)	0.51 (0.47, 0.54)	0.97 (0.95, 0.98)	0.92 (0.87, 0.95)	0.96 (0.94, 0.98)	-0.02 (-0.08, 0.06)
	VinDr-CXR	0.88 (0.83, 0.91)	0.85 (0.84, 0.86)	0.25 (0.23, 0.27)	0.99 (0.99, 0.99)	0.73 (0.68, 0.77)	0.39 (0.37, 0.41)	0.94 (0.93, 0.95)	0.83 (0.78, 0.87)	0.98 (0.95, 0.99)	--
	Shenzhen	0.82 (0.78, 0.85)	0.98 (0.96, 0.99)	0.98 (0.96, 0.99)	0.84 (0.81, 0.87)	0.8 (0.76, 0.85)	0.89 (0.87, 0.92)	0.97 (0.96, 0.98)	0.91 (0.86, 0.96)	0.99 (0.96, 1.0)	--
	Montgomery	0.67 (0.53, 0.78)	1.0 (1.0, 1.0)	1.0 (1.0, 1.0)	0.81 (0.75, 0.85)	0.67 (0.53, 0.78)	0.8 (0.7, 0.87)	0.99 (0.97, 1.0)	0.98 (0.88, 1.0)	1.00 (1.0, 1.0)	--
Abn.	ChestX-ray8	0.82 (0.78, 0.84)	0.86 (0.81, 0.89)	0.94 (0.92, 0.95)	0.65 (0.61, 0.69)	0.68 (0.62, 0.72)	0.87 (0.85, 0.89)	0.92 (0.9, 0.93)	--	--	--
	VinDr-CXR	0.89 (0.87, 0.9)	0.73 (0.72, 0.75)	0.6 (0.59, 0.63)	0.93 (0.92, 0.94)	0.62 (0.59, 0.64)	0.72 (0.7, 0.73)	0.89 (0.88, 0.9)	--	--	--

*Datasets included: HaMLET; VinDr-CXR, an open dataset of chest x-rays with radiologist annotations from 2 major hospitals in Vietnam; Shenzhen, collected from outpatient clinics and collected in collaboration with Shenzhen No.3 People’s Hospital, Guangdong Medical College, Shenzhen, China; Montgomery, chest x-ray dataset collected in Montgomery County, Maryland, in collaboration with the Department of Health and Human Services; ChestX-ray8, hospital-scale chest x-ray database with disease image labels, provided by the National Institutes for Health (NIH).

### Multilabel classification performance

Table 4 shows the performance metrics for our models on Task 3. AUCs ranged from 0.78 (95% CI 0.73, 0.8) in predicting discrete linear opacities to 0.96 (0.65, 0.99) in predicting pleural effusions. With the J operating point, sensitivity and specificity jointly reached 0.8 for four of the abnormalities: infiltrates, cavities, pleural effusions, and volume loss or retraction. For the other abnormalities, sensitivity at that operating point was higher than specificity, sometimes by a large margin (e.g., 0.91 sensitivity and 0.53 specificity for discrete nodules without classification). Because most abnormalities were rare in our data, metrics that depend on prevalence, like PPV and F1, were consistently low, with F1 reaching a maximum of 0.13 (0.10, 0.15) for infiltrates and dipping as low as 0.00 (0.00, 0.01) for hilar adenopathies.

**Table 4:** Classification metrics for our findings-specific model on our internal dataset.

Finding	Sensitivity	Specificity	PPV	NPV	J	F1	AUC	Rel. Prev. Diff.*
Infiltrate	0.91 (0.88, 0.95)	0.81 (0.81, 0.82)	0.07 (0.06, 0.08)	1.0 (1.0, 1.0)	0.73 (0.68, 0.77)	0.13 (0.1, 0.15)	0.93 (0.91, 0.95)	0.02 (-0.15, 0.2)
Reticular Markings	0.93 (0.84, 1.0)	0.63 (0.63, 0.65)	0.01 (0.01, 0.02)	1.0 (1.0, 1.0)	0.56 (0.47, 0.63)	0.02 (0.01, 0.03)	0.86 (0.82, 0.9)	-0.21 (-0.51, 0.3)
Cavity	0.84 (0.5, 1.0)	0.84 (0.83, 0.84)	0.0 (0.0, 0.0)	1.0 (1.0, 1.0)	0.68 (0.33, 0.84)	0.0 (0.0, 0.01)	0.92 (0.78, 0.98)	1.5 (-0.29, 12.0)
Nodule	0.89 (0.75, 1.0)	0.72 (0.71, 0.73)	0.01 (0.01, 0.01)	1.0 (1.0, 1.0)	0.62 (0.48, 0.73)	0.02 (0.01, 0.02)	0.88 (0.84, 0.92)	-0.09 (-0.45, 0.92)
Pleural Effusion	0.92 (0.0, 1.0)	0.93 (0.92, 0.93)	0.01 (0.0, 0.02)	1.0 (1.0, 1.0)	0.84 (0.43, 0.93)	0.02 (0.01, 0.04)	0.96 (0.65, 0.99)	0.17 (-0.5, 3.0)
Hilar Adenopathy	0.69 (0.0, 1.0)	0.79 (0.79, 0.8)	0.0 (0.0, 0.0)	1.0 (1.0, 1.0)	0.49 (-0.2, 0.8)	0.0 (0.0, 0.01)	0.82 (0.44, 0.97)	-0.5 (-1.0, 3.0)
Discrete Linear Opacity	0.86 (0.8, 0.91)	0.6 (0.59, 0.61)	0.04 (0.03, 0.05)	1.0 (0.99, 1.0)	0.46 (0.4, 0.51)	0.08 (0.06, 0.09)	0.78 (0.73, 0.8)	-0.06 (-0.2, 0.17)
Discrete Nodule	0.91 (0.83, 1.0)	0.53 (0.52, 0.55)	0.01 (0.01, 0.01)	1.0 (1.0, 1.0)	0.44 (0.35, 0.53)	0.02 (0.01, 0.02)	0.8 (0.75, 0.84)	-0.09 (-0.4, 0.41)
Volume Loss	0.88 (0.74, 1.0)	0.85 (0.84, 0.86)	0.02 (0.01, 0.02)	1.0 (1.0, 1.0)	0.73 (0.53, 0.85)	0.03 (0.02, 0.04)	0.93 (0.88, 0.97)	0.19 (-0.33, 1.4)
Irreg. Thick Pleural Reaction	0.92 (0.79, 1.0)	0.69 (0.68, 0.7)	0.01 (0.01, 0.01)	1.0 (1.0, 1.0)	0.61 (0.51, 0.7)	0.02 (0.01, 0.02)	0.86 (0.81, 0.91)	0.12 (-0.32, 1.08)
Other	0.78 (0.56, 0.91)	0.68 (0.67, 0.69)	0.01 (0.0, 0.01)	1.0 (1.0, 1.0)	0.46 (0.24, 0.59)	0.01 (0.01, 0.02)	0.82 (0.73, 0.88)	0.22 (-0.21, 1.44)

With the count operating point, the model produced fairly accurate prevalence estimates for a number of the abnormalities, with three absolute relative errors under 10% (2% for infiltrates, -6% for discrete linear opacities, and -9% for both kinds of nodules), and three under 20% (12% for irregular thick pleural reactions, 17% for pleural effusions, and 19% for volume loss or retraction). We note here that operating point selection is crucial for accuracy on this task with relative errors rising by several orders of magnitude when the J operating point was used instead of the count operating point (S2 Table).

### Approximate abnormality localization

**Fig 2:**
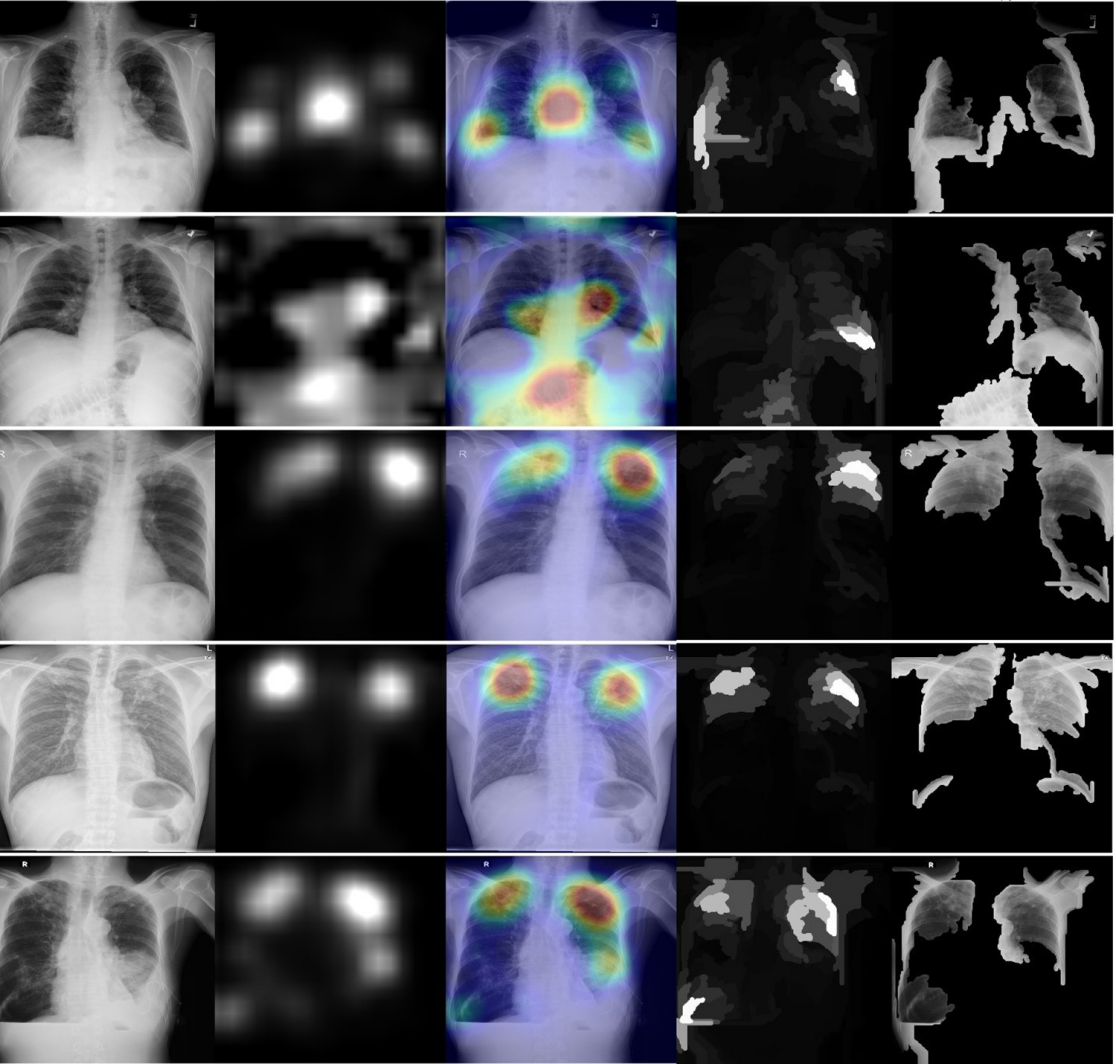
XRAI (left) and GradCAM (right) heatmaps for true positive images. The original radiographs are on the left, the Grad-CAM activations and heatmaps are in the middle, and the XRAI activations and overlays are on the right. For the XRAI overlays, only regions reaching the 70^th^ percentile of activation strength are shown.

**Fig 3:**
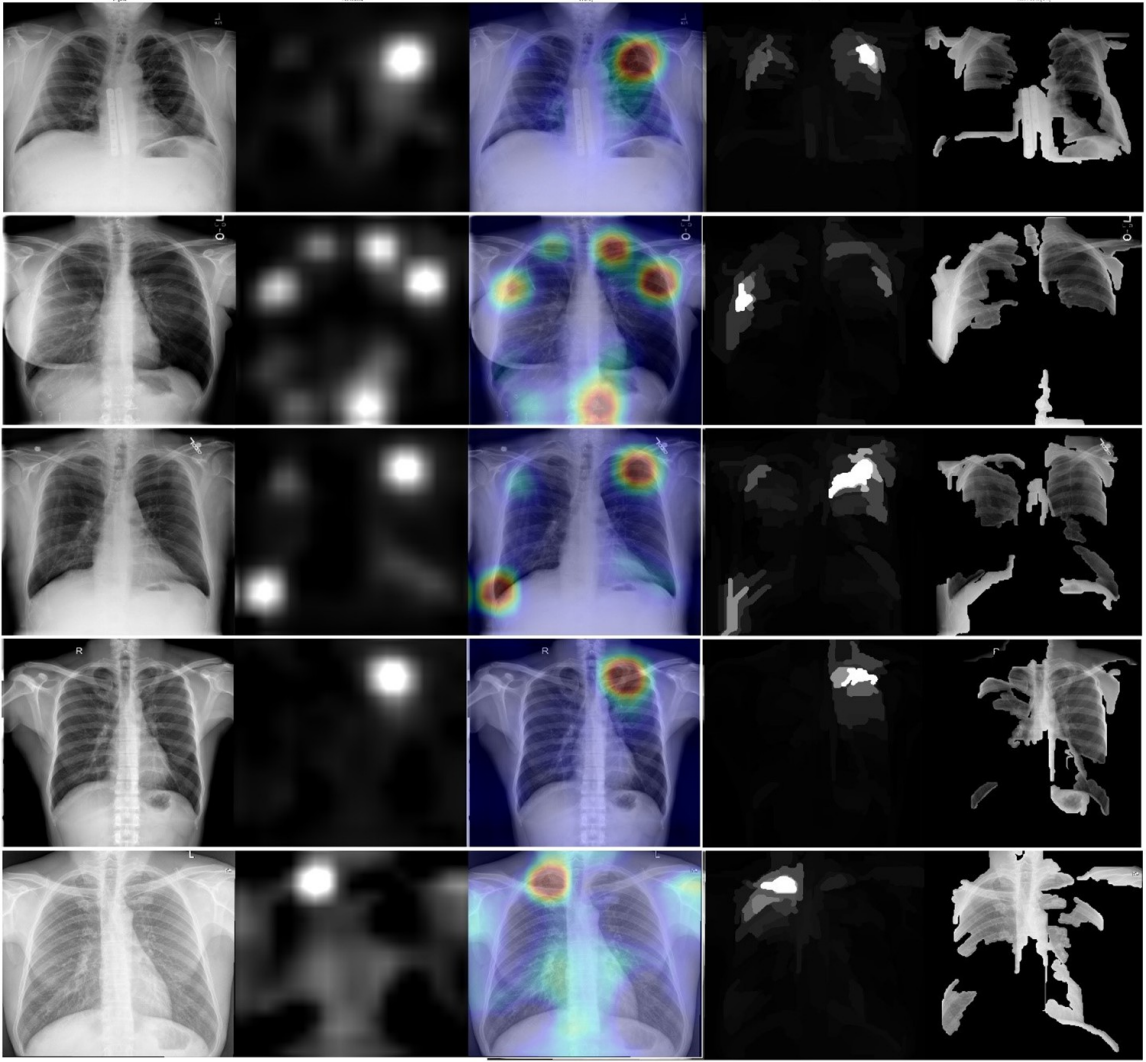
XRAI (left) and GradCAM (right) heatmaps for false positive images. The original radiographs are on the left, the Grad-CAM activations and heatmaps are in the middle, and the XRAI activations and overlays are on the right. For the XRAI overlays, only regions reaching the 70^th^ percentile of activation strength are shown.

Fig 2 shows Grad-CAM (second and third columns) and XRAI (fourth and fifth columns) for five radiographs correctly identified as abnormal by Model 1; the radiographs were drawn from the abnormal examples in ChestX-ray8, SHN, and VinDR-CXR. Fig 3 shows the same panel of heatmaps, but for five images incorrectly identified as abnormal, also by Model 1 and drawn from the same datasets. In general, the two methods identify similar regions of the radiographs as being abnormal, although they do occasionally diverge in both the extent and the severity of the highlighted regions.

## Discussion

### Applications of current models

Our binary models showed strong results on our internal datasets at both of our chosen operating points. Although we do not currently intend for either model to be used for individual-level classification tasks, e.g., as part of the overseas immigration exam clinical workflow, their performance is on-par with top-performing models published in the literature, including several commercial products designed largely to detect radiographic signs of tuberculosis (see Codlin 2021 [42] and Kik 2022 [43]). Performance on the external datasets also suggests that the models may generalize well to unseen data, even on tasks for which we did not have clean validation or testing data, like Task 1, on which our models come within two percentage points of what we believe to be the current state-of-the-art on AUC (0.92 from Model 1 vs. 0.94 from Nabulsi et al. 2021 [23]). The main exception to this trend is Model 3, which despite achieving good AUCs in identifying many of the specific findings, is not likely to be clinically useful, at least in the population represented by our internal data, owing to the rarity of most of the findings and the model’s resulting poor PPV.

A relatively clear use-case for our tuberculosis-specific models (2 and 3), however, is in estimating sample-level counts of abnormal images and, depending on the abnormality in question, specific findings. As a tool for conducting internal QC on radiographs read during the overseas immigration exam, we can imagine running the models on batches of incoming images and comparing their number of positive calls to the number of reported abnormal radiographs, raising an alert when the difference in counts exceeds a predefined threshold and triggering a model-guided manual review for further investigation. In the case of Model 2, for example, which underestimated the number of abnormal images suggestive of tuberculosis in our internal test set by only 2%, a reasonable threshold might be +/- 10%, just beyond the bounds of the 95% CI (-8% to 6%), and the corresponding manual review might begin with the images with the highest model-based probability of abnormality among those initially reported normal. This kind of process may be stratified by key operational variables, like exam site or country, and may also be informed by existing epidemiologic information, like the expected background rates of tuberculosis disease in the screening areas or site-specific historical rates of abnormal radiographs confirmed by earlier QC efforts.

### Directions for future research

In our case, a natural first step for future research would be a follow-up validation study with manual review to explore our models’ utility tools for supporting internal QC efforts. A number of studies have examined the performance of already-trained models, mostly in the form of commercial software, in detecting abnormalities [43] and, in certain cases, tuberculosis disease [26,30]. To our knowledge, ours is the first study to propose evaluating models’ ability to estimate sample-level counts, and more evaluation would be needed before integrating them with existing QC workflows. A designed QC study would also allow for the evaluation of attribution methods, like the saliency heatmaps we produced for Figs 2 and 3, as tools for abnormality localization to assist with manual review, which only a small number of prior studies have rigorously addressed [44–45]. Finally, a designed follow-up study would allow us to examine the model’s performance across relevant clinical, demographic, and geographic subgroups; because the testing images in the current study did not constitute a representative sample of the screening population as a whole, we were unable to do such an examination here, despite the fact that those results would be important to establish before incorporating the model into existing programmatic or clinical workflows.

Similarly, an operational analysis to decide when, where, and how to use the models to improve screening programs would fill a gap in the literature, which to date has focused primarily on examining model performance in clinical contexts rather than the downstream effects of incorporating them into larger workflows. Remaining problems include establishing best practices for selecting operating points by country or facility to optimize detection given local constraints on resources and background rates of tuberculosis; estimating minimum diagnostic performance needed to achieve cost-effectiveness and programmatic efficiency under different operating scenarios; and evaluating the epidemiologic and economic impact of allowing radiologists to use the models for decision support during the overseas screening exams. There is evidence that similar models can improve turnaround time [23] or lower costs [29] associated with diagnostic workflows, and because of the scope of the CDC’s overseas screening program, these seem like potentially fruitful avenues of investigation.

A final direction for future research is in developing and evaluating models for predicting active tuberculosis disease from chest radiographs in combination with relevant clinical, demographic, and immunologic information. To our knowledge, no model in the literature has been trained to predict microbiological or molecular test results alone from the radiograph directly—abnormal radiographs often come from patients with positive test results, but because patients with normal radiographs are not typically tested for TB, the reverse is not necessarily true—and although training datasets may use a mix of radiographic, clinical, and microbiological results as reference standards, models have only been recently developed that accept inputs that are multimodal (i.e., non-radiographic), focusing primarily on relevant information that can be captured in free-text [46].

Given these limitations, for many models, performance in predicting tuberculosis disease is determined primarily by two pieces of information: their performance in identifying images with abnormalities, and the correlation between the presence of those abnormalities and tuberculosis disease in the target population. Occasionally the correlation is high enough for models to meet the TPP, but often it is not, with observed specificities at 90% sensitivity ranging from well over 70% [26] to 60% and below [25] in several well-known commercial algorithms. Using other available information for prediction, whether by stacking an additional model on top of the image-processing module or by altering the module itself to accept multimodal inputs, may yield diagnostic gains, not only because the approach mirrors the process by which clinical diagnoses are made and thus seems *a priori* sensible, but also because patient characteristics like HIV status and history of tuberculosis are known to affect model performance based on the radiographs alone [26,47]. Although some technical innovation may be required to make this approach feasible, it may well improve our ability to predict tuberculosis disease, especially in low-resourced settings where access to trained radiologists is limited, and thus seems well worth pursuing.

## Conclusion

Using data collected from immigrants and refugees during overseas immigration exams prior to entry into the US, we trained and tested three deep learning models for identifying abnormalities on chest radiographs. The models performed well, achieving high scores on our internal test dataset, and nearing state-of-the-art on several external test datasets. We believe the models will be useful for CDC’s internal quality control activities, and their strong performance across datasets suggest they may be useful for a variety of other related tasks.

## Supporting information

S1 Text Supplemental methods. Additional technical information about the data preprocessing, modeling, and analysis behind our primary results.
(DOCX)

S1 Table Exam distribution by year and region. Distribution of overseas health screenings by year and region. Row and column totals are on the margins.
(DOCX)

S2 Table Operating points. Operating points for our internal validation set that optimized three criteria: Youden’s J index (j); the absolute relative error in predicted counts (count); and the same relative but reweighted to take the difference in prevalence for each finding between the validation set and the total available data into account (count_adj). We used only the first and third operating points in our analysis.
(DOCX)
