# Supplementary material for: Development and validation of a deep learning model for detecting signs of tuberculosis on chest radiographs among US-bound immigrants and refugees
Source: PLOS Digit Health. 2024 Sep 30;3(9):e0000612. doi: 10.1371/journal.pdig.0000612 (PMC11441656; doi:10.1371/journal.pdig.0000612)
Supplement: S1 Text — Additional technical information about the data preprocessing, modeling, and analysis behind our primary results. (DOCX) [file pdig.0000612.s001.docx]

**Supplemental Methods**

*Sample size calculation*

We used a simulation procedure to determine the number of abnormal images we would need in our validation and test sets to accurately estimate sensitivity, specificity, and other key performance metrics for our models. As a starting point, we assumed the models would achieve a sensitivity and specificity of 80% for the binary classification tasks, which is roughly in line with similar models in the literature (see main manuscript for examples). The estimation procedure itself followed these steps:

1. Choose a sample size *N_s_*. In our simulation, these ranged from 100 to 15,000.
2. Generate a single random variate *k_abn_* from a binomial distribution with *n* = *N*_s_ and *p* = *p*_abn_, where *p­*_abn_ is the expected prevalence of abnormal images suggestive of TB (in our case, this was 7%).
3. Generate random variates for true positives (TP), false positives (FP), false negatives (FN), and true negatives (TN) with a single draw from a multinomial distribution, where *n* = *N_s_* and *p_k_* is the probability of success for each category *k* as determined by the prespecified levels *p*_se_ and *p*_sp_ of sensitivity and specificity, respectively. Explicitly, these are:

*P_TP_*  = *p*_se_ * *p*_abn_

*p_TN_* = *p*_sp_ * (1 – *p_abn_*)

*p_FP_* = 1 – *p_TP_*

*p_FN_* = 1 – *p_TN_*

1. Repeat steps 1 and 2 a large number of times (in our case 1,000) and record the full spectrum of performance metrics based on the resulting confusion matrices.
2. Record the 2.5^th^ and 97.5^th^ percentiles for each metric, and take the half-width of the resulting interval as the statistical precision in estimating the metric at the given sample size *N*_s_.
3. Increase sample size by 100 and repeat steps 2 through 6 until the maximum sample size has been exceeded.

We aimed for a minimum sample size that would allow us to calculate sensitivity and specificity to within 5%. Both our validation and test sets exceeded this minimum by a large margin with an approximate precision for each metric of 1.25%.

*Image preprocessing*

We began by excluding from our datasets radiographs with corrupt DICOM files or corrupt pixel arrays. For DICOM files without such corruptions, we then extracted their raw pixel arrays, converted them to grayscale, and exported them to 1,024x1,024-pixel PNG files. Once the image files were saved, we ran a separate script that used optical character recognition (OCR) software (detailed in *Software and hardware* below) to identify radiographs with burned-in annotations and discarded any identified as containing more than three words of text. This exclusion step served mainly to protect the identities of the entrants by removing personally identifiable information (PII) from the images, but it also prevented the text information from affecting the model’s performance. Finally, we discarded images with mean pixel values (out of 255) below 50 or above 215 which were either too dark or too light, respectively, for radiographic features to be visible.

*Model architecture*

To improve the model’s ability to generalize to unseen data, we used a custom image augmentation layer as the input layer with the following transformations and deltas:

Horizontal flips (0.5 probability);

Resizing (either 0.7x, 0.85x, 1.15x, or 1.3x the original dimensions);

Changes in brightness (delta range -0.525 to 0.525);

Changes in contrast (delta range 0.349 to 1.346);

Changes in saturation (delta range 0.382 to 1.403); and

Changes in hue (delta range -0.127 to 0.127).

The delta ranges were taken from Majkowska 2020 and did not vary during training, and the transformations were turned off during validation and testing. The (sometimes perturbed) images were then passed to the EfficientNetV2M feature extractor for processing, after which they passed through a dropout layer (p=0.5) and then a randomly initialized final dense layer with either binary or categorical cross-entropy loss, depending on the task, for final classification.

*Model training and validation procedures*

We trained all models with a minibatch size of 12 (4 per GPU), which allowed us to fine-tune all blocks of the feature extractor at once (in larger minibatches, or on GPUs with lower amounts of memory, the blocks may need to be unfrozen sequentially to prevent out-of-memory errors). We trained each model until its AUC on the validation data began to decrease; in our case, this yielded one epoch of training for the abnormal/normal model (Model 1), two for the abnormal-TB model (Model 2), and two for the multilabel model (Model 3). In all cases, we used the Adam optimizer (Kingma 2014) with a fixed learning rate of 0.001.

*Operating point selection*

When validation data was available, we used it to select two operating points for thresholding a model’s predictions on the test data: one that maximized Youden’s J index and one that minimized the model’s error in predicting the positive samples. We calculated the first directly from the model’s predicted probabilities on the validation data, but we calculated the second by reweighting the positive samples so that test data prevalence equaled the prevalence in the entire dataset before splitting (they were up-weighted in the validation and test sets to improve precision in estimating sensitivity). Our reweighting method was informed by the following observations:

1. Sensitivity (true positive rate, or TPR) and specificity (1 minus the false positive rate, or FPR) are conditional on the true label (e.g., abnormality or the presence of a specific abnormality) and thus independent of prevalence.
2. True prevalence and predicted prevalence are equal when the false positive rate (FPR) and false negative rate (FNR) are equal.
3. When FPR and FNR are equal, the number of counts in the off-diagonal cells of the confusion matrix are equal (this is the mathematical insight behind McNemar’s test for the difference in paired proportions).
4. The off-diagonal cells are equal to the expressions FPR * (1 – *p*) and FNR * *p*, respectively, where *p* is equal to true prevalence.
5. FNR is equal to 1 – sensitivity, and FPR is equal to 1 – specificity.
6. The ROC curve for a given classifier on a test set contains all possible pairs of sensitivity and specificity.
7. The relative difference in prevalence for any given tuple of TPR, FPR, and *p* is given by the expression |[FPR * (1 – *p*)] – [FNR * *p*]| / *p*, where FPR = 1 – specificity and FNR = 1 – sensitivity.

To select the second operating point, then, we simply used the FPR, TPR pairs from a model’s ROC curve on the validation data to calculate the difference between true prevalence and predicted prevalence given a target true prevalence *p_t_* for each possible operating point (using the formula from observation 4 above) and selected the one with the smallest difference.

*Software and hardware*

All models were built with Keras using the TensorFlow 2 backend. DICOM work was done with the pydicom Python package; image preprocessing with the pytesseract, scikit-image, and NumPy packages; data visualization with the seaborn package; and statistical analysis with custom functions for bootstrap resampling. All code is publicly available at <https://github.com/cdcai/hamlet.git>, and top-level scripts are designed to be rerun on different datasets to aid replication of our results and future research efforts.

Models were trained on a scientific workstation with 32 logical processors, 128GB of RAM, and 3 NVIDIA RTX A6000 GPUs.

*Bootstrap resampling procedures*

To create confidence intervals for the performance metrics, we used the nonparametric bootstrap with bias correction and acceleration (BCA), following Efron 1987. Because the test set contained an even number (4,000) of normal and abnormal images each (we up-sampled abnormal images to increase precision in estimating AUROC and sensitivity), naively constructing the intervals would yield inflated estimates for metrics like positive predictive value (PPV), F1-score, and the relative difference in prevalence that are affected by the underlying prevalence of the abnormal images. To account for this inflation, we constructed each bootstrap replicate by sampling n_ab_ abnormal images from the test set, where n_ab_ is a random draw from a binomial distribution B(n, p), n is the total number of images in the test set (8,000), and p is the proportion of abnormal images in the training data. Each replicate was then filled out with 1 – n_ab_ normal images, also sampled from the test data with replacement, and then the replicate was used to calculate the full range of performance metrics, including sensitivity and specificity (although these are not affected by prevalence). We used this sampling distribution to generate the bias correction parameters z0 and acceleration parameters for the metrics and construct the resulting 95% confidence intervals. As noted in the main manuscript, we did not adjust the intervals for multiplicity.

*References*

Efron B. Better bootstrap confidence intervals. *Journal of the American Statistical Association*. 1987 Mar 1;82(397):171-85.

Majkowska A, Mittal S, Steiner DF, Reicher JJ, McKinney SM, Duggan GE, Eswaran K, Cameron Chen PH, Liu Y, Kalidindi SR, Ding A. Chest radiograph interpretation with deep learning models: assessment with radiologist-adjudicated reference standards and population-adjusted evaluation. *Radiology*. 2020 Feb;294(2):421-31.
