# Supplementary material for: Development and validation of a deep learning model for detecting signs of tuberculosis on chest radiographs among US-bound immigrants and refugees
Source: PLOS Digit Health. 2024 Sep 30;3(9):e0000612. doi: 10.1371/journal.pdig.0000612 (PMC11441656; doi:10.1371/journal.pdig.0000612)
Supplement: S1 Table — Distribution of overseas health screenings by year and region. Row and column totals are on the margins. (DOCX) [file pdig.0000612.s002.docx]

|  |  | **Exam Region** | | | | | |  |
| --- | --- | --- | --- | --- | --- | --- | --- | --- |
|  |  | Africa | Americas | Asia | Europe | Oceania | N/A |  |
| **Exam Year** | 2011 | 0 | 3 | 0 | 0 | 0 | 0 | 3 |
|  | 2012 | 0 | 26 | 0 | 0 | 0 | 0 | 26 |
|  | 2013 | 0 | 75 | 170 | 0 | 0 | 0 | 245 |
|  | 2014 | 0 | 27 | 275 | 0 | 0 | 0 | 302 |
|  | 2015 | 66 | 54 | 774 | 79 | 0 | 0 | 973 |
|  | 2016 | 217 | 91 | 846 | 88 | 0 | 2 | 1244 |
|  | 2017 | 173 | 127 | 928 | 83 | 0 | 3 | 1314 |
|  | 2018 | 709 | 694 | 1388 | 196 | 58 | 0 | 3045 |
|  | 2019 | 8826 | 8794 | 18298 | 4038 | 349 | 78 | 40383 |
|  | 2020 | 10010 | 15246 | 31419 | 6006 | 613 | 186 | 63480 |
|  | 2021 | 9752 | 18159 | 23657 | 4879 | 433 | 116 | 56996 |
|  |  | 29753 | 43296 | 77755 | 15369 | 1453 | 385 |  |
|  | | | | | | | | |
