# Supplementary material for: Development and validation of a deep learning model for detecting signs of tuberculosis on chest radiographs among US-bound immigrants and refugees
Source: PLOS Digit Health. 2024 Sep 30;3(9):e0000612. doi: 10.1371/journal.pdig.0000612 (PMC11441656; doi:10.1371/journal.pdig.0000612)
Supplement: S2 Table — Operating points for our internal validation set that optimized three criteria: Youden’s J index (j); the absolute relative error in predicted counts (count); and the same relative but reweighted to take the difference in prevalence for each finding between the validation set and the total available data into account (count_adj). We used only the first and third operating points in our analysis. (DOCX) [file pdig.0000612.s003.docx]

| **Finding** | **j** | **count** | **count_adj** |  |  |  |
| --- | --- | --- | --- | --- | --- | --- |
| abnormal | 0.15459 | 0.147612 | 0.448105 |  |  |  |
| abnormal_tb | 0.056503 | 0.037703 | 0.355039 |  |  |  |
| infiltrate | 0.042922 | 0.317821 | 0.860177 |  |  |  |
| reticular | 0.020081 | 0.381927 | 0.530253 |  |  |  |
| cavity | 0.098012 | 0.358573 | 0.773157 |  |  |  |
| nodule | 0.063748 | 0.388444 | 0.890132 |  |  |  |
| pleural_effusion | 0.056165 | 0.736675 | 0.998791 |  |  |  |
| hilar_adenopathy | 0.024143 | 0.328604 | 0.922602 |  |  |  |
| linear_opacity | 0.036351 | 0.371436 | 0.612073 |  |  |  |
| discrete_nodule | 0.008291 | 0.366458 | 0.754047 |  |  |  |
| volume_loss | 0.052816 | 0.305774 | 0.853881 |  |  |  |
| pleural_reaction | 0.028239 | 0.298953 | 0.809463 |  |  |  |
| other | 0.010258 | 0.203865 | 0.672526 |  |  |  |
|  |  |  |  |  |  |  |
|  | | | | | | |
